# STING Signaling in Cancer Cells: Important or Not?

**DOI:** 10.1007/s00005-017-0481-7

**Published:** 2017-07-26

**Authors:** Olga Sokolowska, Dominika Nowis

**Affiliations:** 1 Laboratory of Experimental Medicine, Centre of New Technologies, University of Warsaw, Banacha 2c, 02-097 Warsaw, Poland; 2 Department of Immunology, Center of Biostructure Research, Medical University of Warsaw, Warsaw, Poland; 3 Postgraduate School of Molecular Medicine, Medical University of Warsaw, Warsaw, Poland; 4 Genomic Medicine, Medical University of Warsaw, Warsaw, Poland; 5 Centre for Preclinical Research and Technology, Medical University of Warsaw, Warsaw, Poland

**Keywords:** STING, Type I interferons, Innate immunity, Cancer immunology, Cyclic dinucleotides

## Abstract

Stimulator of interferon genes (STING) is an adaptor protein that plays an important role in the activation of type I interferons in response to cytosolic nucleic acid ligands. Recent evidence indicates involvement of the STING pathway in the induction of antitumor immune response. Therefore, STING agonists are now being extensively developed as a new class of cancer therapeutics. However, little is known about the consequences of activated STING-mediated signaling in cancer cells on the efficacy of the antitumor treatment. It has been shown that activation of the STING-dependent pathway in cancer cells can result in tumor infiltration with immune cells and modulation of the anticancer immune response. Understanding the function of STING pathway in cancer cells might provide important insights into the development of effective therapeutic strategies. This review focuses on the role of STING pathway in cancer cells, the largely unknown topic that has recently emerged to be important in the context of STING-mediated antitumor responses.

## Introduction

Innate immunity is a critical component of host defense against various pathogens, such as viruses, bacteria, fungi and parasites. Its functioning is based on the recognition of pathogen-associated molecular patterns (PAMPs) or danger-associated molecular patterns (DAMPs) through a set of pattern recognition receptors that stimulate the downstream signaling cascades leading to production of proinflammatory mediators and type I interferons (IFNs) (Takeuchi and Akira 2010). Exogenous DNA derived from pathogens or self-DNA in the cytosol are powerful PAMPs/DAMPs for which host organism possesses the DNA sensing systems with many DNA sensors and downstream adaptors to induce innate immune responses (Paludan and Bowie 2013). Within this system stimulator of interferon genes (STING) protein was shown to be a critical mediator of the signaling triggered by cytosolic nucleic acid derived from DNA viruses and bacteria (Ishikawa and Barber 2008; Ishikawa et al. 2009). The ability of STING to induce the production of type I IFNs drove scientists to explore this pathway in the context of antitumor immune response (Woo et al. 2014; Zhu et al. 2014). Recently, STING has emerged to be a potent target of anticancer therapies (Chandra et al. 2014; Deng et al. 2014; Ohkuri et al. 2014). It was immediately suggested that the majority of the antitumor effects caused by STING activation depend upon production of IFN-β by antigen-presenting cells (APCs) that promotes CD8^+^ T cell priming against tumor-associated antigens (Klarquist et al. 2014; Woo et al. 2014). However, STING protein is expressed broadly in a variety of cell types including cancer cells, in which the function of the pathway has not been well characterized.

## STING Pathway

**Fig. 1:**
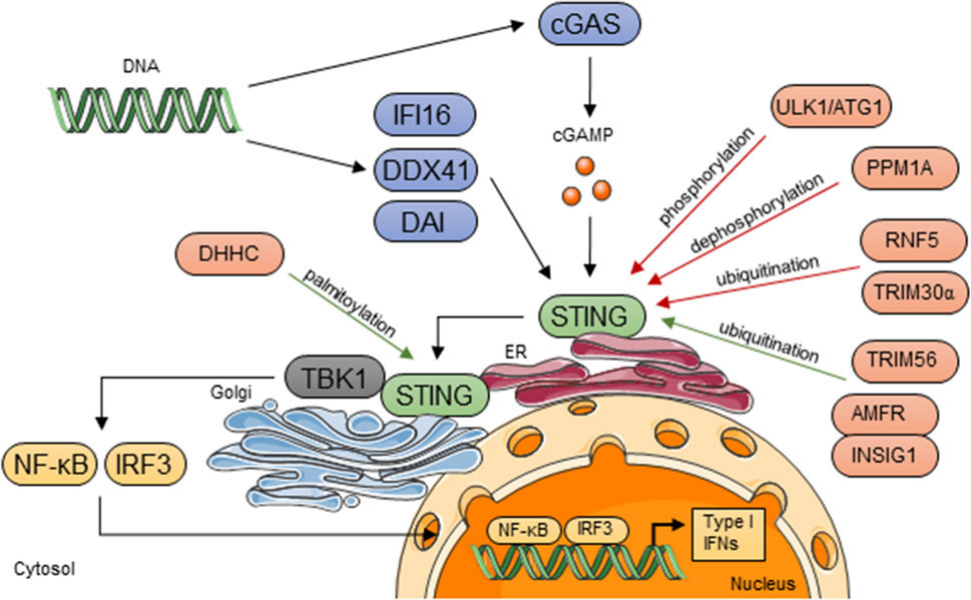
STING-dependent innate immune signaling. STING is activated by cGAMP produced by cGAS or by other DNA sensors DAI, IFI16, DDX41 after detection of DNA in cytoplasm. Upon activation STING translocates from the ER to perinuclear Golgi compartments, where it interacts with TBK1. TBK1 phosphorylates and activates transcription factors IRF3 and NF-κB, which translocate to nucleus to promote transcription of type I IFNs genes. Activation of STING is regulated by different post-translational modifications, including phosphorylation, ubiquitination and palmitoylation by enzymes like kinase ULK1/ATG1, phosphatase PPM1A, ubiquitin ligases RNF5, TRIM30α and TRIM56, AMFR and INSIG1, palmitoyltransferases DHHC3, DHHC7 and DHHC15. *Red arrows* depict modifications that suppress, and *green arrows*, those that promote STING-dependent signaling. The figure was prepared using Servier Medical Art (http://www.servier.com)

STING (also known as MITA, MPYS, ERIS and TMEM173) is an ubiquitously expressed adaptor protein localized predominantly on the endoplasmic reticulum (ER) membrane, where it is anchored through several transmembrane domains residing in its N-terminal region (Ishikawa and Barber 2008). It is encoded by *TMEM173* gene, which in humans can have up to five variants including the wild-type allele, the reference allele (R232H), the HAQ allele (R71H, G230A, R293Q), the AQ allele (G230A, R293Q), and the Q allele (R293Q), that differ in the ability to induce downstream signaling (Yi et al. 2013). In addition, the transcript can undergo the alternative splicing that results in generation of isoform lacking C-terminal domains, acting as a dominant negative regulator of STING-mediated induction of type I IFN response (Chen et al. 2014). The model of STING activation is controversial. Some studies suggest that without stimulation STING exists as a monomer that is activated by dimerization when stimulated (Sun et al. 2009; Tsuchida et al. 2010). Other studies suggest that STING forms dimers in the absence of stimulation (Ouyang et al. 2012; Shu et al. 2012). Yin et al. (2012) proposed an alternative model of activation where STING forms a dimer; however, it is inactive without stimulation due to an inhibitory interaction between the carboxyl terminal tail and the carboxyl binding domain. This interaction is disrupted upon stimulation enabling the interaction with the downstream effectors (Yin et al. 2012). STING can be activated upon detection of DNA in cytosol by different DNA sensors such as DNA-dependent activator of interferon regulatory factors (DAI), IFN-γ-inducible protein 16 (IFI16), DEAD box polypeptide 41 (DDX41) (Wu and Chen 2014). However, the most critical receptor for this pathway is cyclic GMP-AMP (cGAMP) synthase (cGAS) that produces cyclic dinucleotides referred to as 2′3′-cGAMP, which can bind STING directly (Ablasser et al. 2013; Civril et al. 2013; Sun et al. 2013). Activated STING translocates from the ER through the Golgi apparatus to the perinuclear microsomal compartments in a mechanism dependent on autophagy-related protein Atg9a (Ishikawa et al. 2009; Saitoh et al. 2009). Its translocation to the Golgi is required for the correct function of the STING pathway since the disruption of this process with brefeldin A treatment or expression of Shigella effector IpaJ leads to the abrogation of the downstream signaling (Ishikawa et al. 2009; Mukai et al. 2016). When STING reaches the Golgi, it forms aggregates driving activation of tank-binding kinase 1 (TBK1) that subsequently phosphorylates STING. This post-translational STING modification results in the recruitment of the interferon regulatory factor 3 (IRF3) to the complex and its phosphorylation by TBK1 (Liu et al. 2015a; Tanaka and Chen 2012). After phosphorylation IRF3 translocates to the nucleus and triggers transcription of *IFNB1* and several other genes, which promote expression of proinflammatory cytokines, such as interleukin 6 and tumor necrosis factor α (Ishikawa et al. 2009; Woo et al. 2014). TBK1 also activates the NF-κB pathway by phosphorylation of IKKαβ (Abe and Barber 2014) (Fig. 1).

## Post-translational Regulation of the STING Pathway

Acute, self-resolving inflammation in response to infection is crucial for pathogen elimination by the host; however, prolonged, chronic inflammation often leads to the development of immunopathology. Aberrant STING signaling was shown to be involved in the development of autoimmune diseases like Aicardi–Goutières syndrome or severe systemic lupus erythematosus (Ahn et al. 2014; Gall et al. 2012). Thus, STING activity has to be tightly regulated. One of most important post-translational modifications of STING is its phosphorylation. TBK1-mediated phosphorylation of serine 358 and 366 is essential for the triggering of STING pathway signaling (Liu et al. 2015a; Zhong et al. 2008). However, the same serine 366 residue is also phosphorylated by serine/threonine UNC-51-like kinase (ULK1/ATG1) to facilitate the degradation of STING. Moreover, the removal of phosphate group of serine 358 by Mg^2+^/Mn^2+^-dependent protein phosphatase 1A (PPM1A) was identified as a negative regulator of the pathway (Li et al. 2015). Another protein modification that can either promote or suppress STING-dependent signaling is ubiquitination. K48-linked ubiquitination catalyzed by ubiquitin ligase RNF5 or TRIM30α serves as a signal for proteasome-mediated STING degradation (Wang et al. 2015; Zhong et al. 2009). At the same time K63-linked ubiquitination by TRIM56 induces STING dimerization (Tsuchida et al. 2010) and K27-linked ubiquitination mediated by autocrine motility factor receptor (AMFR) and insulin-induced gene 1 (INSIG1) is indispensable for STING interaction with TBK1 (Wang et al. 2014). STING has not been identified as a target for N-linked glycosylation (Tang et al. 2016) but was shown to be palmitoylated at several cysteine residues including C88 and C91. Suppression of the palmitoylation abolishes transcription of downstream genes upon activation of the pathway (Mukai et al. 2016). Moreover, other proteins involved in the STING pathway, such as cGAS or IRF3, undergo post-transcriptional modifications (Du et al. 2015; Hu et al. 2016) adding more complexity to its already elaborate regulatory mechanisms.

## STING Pathway in Cancer Cells

Although STING expression is usually suppressed or lost in the majority of cancers, it is detected at various levels in some tumors (Bhatelia et al. 2014; Kodigepalli and Nanjundan 2015; Xia et al. 2016a). For example, in a murine pancreatic cancer model STING is expressed not only in stromal cells but also in cancer cells themselves (Baird et al. 2016). STING protein has also been detected in human pancreatic ductal adenocarcinomas (Baird et al. 2016). Immunohistochemical staining and RNA analysis of paraffin-embedded samples from patients with colon adenocarcinoma showed that STING is expressed in cancer cells, and that there is a significant loss of the expression at later stages of the disease starting from stage II, with more profound losses in the most advanced lesions (Xia et al. 2016a). A similar, tumor stage-related suppression of STING expression has been reported in malignant melanoma samples with the loss of STING expression in 23.2% of early malignant melanoma samples and 41.7% in more advanced, metastatic disease (Xia et al. 2016b). STING expression was also down-regulated in human papillomavirus (HPV) positive low grade squamous intraepithelial lesions compared with HPV negative controls (Sunthamala et al. 2014). Contrary results were obtained in tongue squamous cell carcinoma samples where no differences in STING expression between HPV-positive and HPV-negative samples were found, but there was a significant increase of STING expression in cancer cells in comparison with normal tissue (Liang et al. 2015). Expression of other proteins involved in the STING pathway can also be suppressed in cancer cells. The expression of mRNA encoding IFI16, STING, TBK1, and IFN-β was significantly decreased in the colorectal cancer tissues in comparison to normal tissue in a cohort of Chinese colorectal cancer patients (Yang et al. 2017). These data strongly suggest cancer type-specific differences in STING expression. Moreover, it seems that the suppression of STING expression occurs more commonly than its upregulation, especially in more advanced diseases.

The presence of STING protein in cancer cells allows investigating the outcomes of its activation by STING agonists, including cGAMP (Tang et al. 2016) or dsDNA (Xia et al. 2016a). Such studies revealed that the function of the pathway and the ability to activate downstream proteins and genes is often defective in tumors cells. Xia et al. (2016a) tested 11 human colorectal adenocarcinoma cell lines and observed that dsDNA treatment led to low level of IFN-β production, that was lower than in normal colon epithelial cells. Moreover, only four cell lines showed STING trafficking or phosphorylation after incubation with dsDNA. None of the evaluated cell lines had major mutation in STING or cGAS encoding genes; however, five cell lines did not express cGAS partially due to epigenetic modifications (Xia et al. 2016a). Similar results were obtained recently by the same group in the melanoma model (Xia et al. 2016b).

Inhibition of the STING pathway is also observed in cancer cells in the process of viral oncogenesis mediated by the DNA oncoviruses. For example, viral oncogenes such as HPV E7 protein and adenoviral E1A disrupt the STING pathway by direct binding to STING protein. This effect was reversed when oncogenes were silenced (Lau et al. 2015). Also vIRF1, which is a protein encoded by Kaposi’s sarcoma-associated herpesvirus (KSHV, HHV8) and NS4B, a hepatitis C virus protein, interact directly with STING and prevent its binding to TBK1, leading to inhibition of the STING signaling (Ding et al. 2013; Ma et al. 2015). Moreover, hepatitis B virus polymerase inhibits STING K63-linked ubiquitination, which causes the loss of STING function (Liu et al. 2015b). Some viral oncogenes can bind to other proteins involved in the STING pathway. Human T cell leukemia virus type 1 oncoprotein Tax interacts with TBK1 rendering it unable to phosphorylate IRF3 (Yuen et al. 2016), while HPV-E6 oncogene can bind directly to IRF3 and inhibit its transcriptional activity (Ronco et al. 1998). It is not surprising that oncoviruses employed various strategies to abolish the STING pathway since it is a major innate immune pathway involved in antiviral response. Inhibition of the signaling allows viruses to avoid elimination by the immune system. However, it is currently unclear if oncoviruses-mediated inhibition of the STING pathway in tumor cells is merely a result of virus-induced evasion of the host immune responses, or is an additional event participating in tumorigenesis.

The influence of STING-mediated signaling on cancer cells is not clear since the results from various studies are to some extent conflicting. Knocking out of *TMEM173* (the gene encoding STING) did not affect B16D8 melanoma cell line proliferation or survival (Takashima et al. 2016). Furthermore, STING agonists did not show any cytotoxic effects against murine cancer cell lines, including B16F10 melanoma, SCCFVII upper aero-digestive squamous cell carcinoma (Fu et al. 2015), CT26 colon carcinoma (Li et al. 2016), Hepa 1-6 hepatoma, LL/2 Lewis lung cancer (Tang et al. 2016), or human HSC-3 and Scc-4 tongue squamous cell carcinomas (Liang et al. 2015). However, several other studies indicated that STING agonists can induce cancer cell death directly. Chandra et al. (2014) observed that 4T1 breast cancer cells have increased levels of activated caspase 3 and reduced viability after incubation with serial dilutions of c-di-GMP. Moreover, overexpression of STING in MCF-7 or T47D breast cancer cell lines with low basal expression of this protein caused an increase in caspase 3 and/or 7 activity, which led to higher rates of cell death (Bhatelia et al. 2014). Tang et al. (2016) hypothesize that cytotoxic effects of STING activation are limited to B cell-derived malignancies. They showed that activation of STING with 3′3′-cGAMP in primary B-cell chronic lymphocytic leukemia cells isolated from Eµ-TCL1 mice, A20 murine B-cell lymphoma, and 5TGM1 murine multiple myeloma induce mitochondria-mediated apoptosis. The effect was dose-dependent and was not caused by increased expression of type I IFNs (Tang et al. 2016). Different results were obtained by Gaston et al. (2016) who showed that in vitro treatment with genotoxic agent mafosfamide induced IFN signaling only in selected breast cancer cell lines including MCF7 and a patient-derived xenograft primary cell line. The treatment resulted in significant STING-dependent increase in expression of genes encoding type I IFNs as well as secretion of type I IFNs into the culture medium. Interestingly, while the chemotherapy-induced secretome of MCF7 cells reduced the viability of treatment naïve cancer cells, the activation of the STING pathway in breast cancer cells made them more resistant to genotoxic stress caused by mafosfamide (Gaston et al. 2016). This shows the opposite biological effects of the STING pathway activation in cancer cells that need further clarification. This study provides further evidence on the complexity of the STING pathway function in cancer cells and it clearly demonstrates that within the same cancer type cell lines can respond differently to STING activation. Further work is needed to determine if STING activation in cancer cells can lead to cancer cell death and if this effect is specific for given type of malignancy. It should be also noted that in vivo meaning of the STING pathway activation in cancer cells might be affected by the influence of the tumor microenvironment.

## STING Pathway in Cancer Cells and the Development of Antitumor Immune Response

It may be beneficial for cancer cells to downregulate STING-mediated signaling in order to avoid immune destruction. It was shown that in murine and human lymphoma cell lines the DNA damage response (DDR) is a source of cytosolic DNA that induces expression of retinoic acid early transcript (RAE1), an NKG2D ligand, in a mechanism dependent on the STING pathway. Knockdown of either of following genes: *TMEM173*, *TBK1* and *IRF3* with specific shRNA led to reduced expression of RAE1 after induction of DNA damage by cytosine b-d-arabinofurano-side hydrochloride (Ara-C) in BC2 cell line or downregulation of constitutive RAE1 expression in Yac-1 cell line (Lam et al. 2014). Interaction of NKG2D ligands with NKG2D receptor located on NK cells regulates their activation and induction of antitumor response (Guerra et al. 2008), therefore inhibition of the STING pathway can facilitate the escape of cancer cells from immunosurveillance by decreasing their capacity to activate NK cells. This hypothesis was further supported by studies performed by Takashima et al. (2016), who reported that STING in B16D8 melanoma cells is involved in NK cell recruitment to the tumor site and their activation. Using STING knock out cell line they showed that STING deficiency in melanoma cells leads not only to a faster tumor growth but also to reduced infiltration with NK cells in STING-deficient mice. The levels of NK cells activation products, including IFN-γ, granzyme B and perforin, were also downregulated. The absence of STING in cancer cells influenced also cytokine secretion, causing decrease in production of CCL5 and CXCL10 that are involved in NK cell activation and infiltration into tumor (Takashima et al. 2016). Additionally, the role of STING pathway in the induction of CD4^+^ and CD8^+^ lymphocytic infiltration into tumor is now emerging. In DDR-deficient molecular subtype of breast cancer that is associated with loss of the FA/BRCA pathway, the number of CD4^+^ and CD8^+^ T cells is significantly higher than in tumors with functional DNA damage response system. It was shown to be a result of the STING pathway-mediated increase in production of cytokines that are crucial for chemotaxis of CD4^+^ and CD8^+^ T cells—CCL5 and CXCL10 (Parkes et al. 2017). Furthermore, the STING-dependent pathway was reported to be involved in immune rejection of prostate cancer cells in mice. TRAMP-C2 murine prostate cancer cells, with impaired STING expression acquired with CRISPR-Cas9 technology, were characterized by a lower rate of rejection in short-term rejection assays in comparison with their wild-type counterparts. The rate of phagocytosis of those cells was also decreased (Ho et al. 2016). In summary, the data suggest that in cancer cells the STING pathway can be important for inducing the antitumor immune response and downregulation of STING expression can facilitate avoiding immune destruction.

**Fig. 2:**
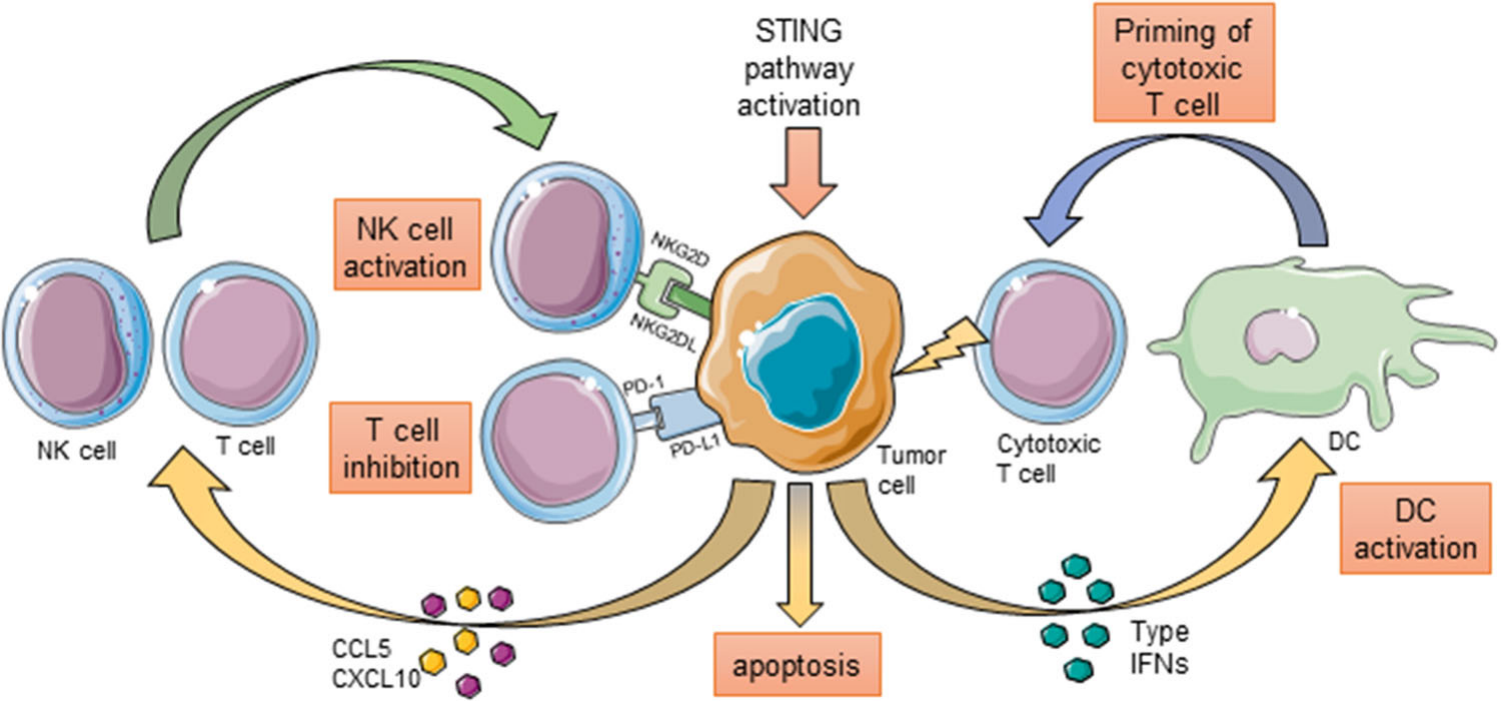
Possible effects of activation of STING signaling in tumor cells. Activation of STING pathway in tumor cells can lead to secretion of chemokines CCL5 and CXCL10 that promote infiltration of NK cells and T cells into tumor. STING-mediated signaling induces expression of ligands NKG2DL on tumor cells that participate in activation of NK cells and induction of antitumor response. On the other hand it promotes also the expression of PD-L1 that plays a major role in suppressing the function of T cells. Cancer cells can also secrete type I IFNs that activate DCs to facilitate the priming of cytotoxic T cells. STING pathway activation may also lead to cancer cell death. The figure was prepared using Servier Medical Art (http://www.servier.com)

On the other hand activation of STING can also have the immunosuppressive effects through the upregulation of programmed death-1 ligand (PD-L1). Interaction of PD-L1 with PD-1 leads to inhibition of the activation of T cells and therefore has a crucial role in tumor immune escape (Wang et al. 2016). Incubation of Panc02 cells with STING agonist led to upregulation of MHCI and PD-L1 expression (Baird et al. 2016). Also, the treatment of murine tumor models with STING agonist caused increase in PD-L1 expression on tumor cells (Fu et al. 2015). Recently it was shown that STING-mediated signaling is required for upregulation of PD-L1 in breast cancer in response to DNA damaging agents like cisplatin (Parkes et al. 2017) (Fig. 2).

However, when considering the role of STING-mediated signaling in the development of antitumor immune response, it is worthwhile remembering that the STING pathway is activated strongly in APCs and to a lesser extent in stromal cells and T cells, and that in fact the APCs play a critical role in the in vivo antitumor effects of STING agonists treatment (Corrales et al. 2015).

## Therapeutic Potential

Oncolytic virotherapy is an emerging anticancer treatment modality that uses engineered viruses to selectively destroy cancer cells. In 2015 the first live virus—talimogene laherparepvec (T-Vec) was approved by the US Food and Drug Administration for the treatment of melanoma (Grigg et al. 2016). Many oncolytic viruses are currently developed and tested in clinical trials; however, their efficacy is still not satisfactory (Russell et al. 2012). It was shown that melanoma and colorectal cancer cells with defective STING-mediated signaling are more susceptible to oncolytic virotherapy (Xia et al. 2016a, b). In nude mice the intratumoral administration of a virus decreased growth of STING pathway defective tumors, while tumors with partial pathway activity were refractory to the treatment. This effect is likely caused by the inability of cells with a completely defective STING signaling to induce antiviral response, which results in robust viral replication and in consequence rapid cancer cell death (Xia et al. 2016a, b).

While oncolytic viruses are more effective in cancer cells without STING expression, PD-1 blockade treatment can turn out to be more effective in STING-positive tumors. Tumors from mice treated with a STING agonist show increased levels of PD-L1 and tumor-infiltrating CD8^+^IFN-γ^+^ T cells. Combination treatment of STING agonist and anti-PD-1 antibody in CT26 and B16 murine tumor models induced regression of tumors that did not respond to the checkpoint inhibition alone (Fu et al. 2015).

The rationale for combining STING agonists with DNA damaging agents needs to be further verified since it is not known if the increased resistance of cancer cells to genotoxic stress caused by the STING pathway activation (Gaston et al. 2016) will overcome the significance of antitumor immune response induction related to increased expression of NKG2D ligand (Lam et al. 2014) and secretion of cytokines that facilitate tissue repair and antitumor-T cell priming (Gaston et al. 2016; Xia et al. 2016a). What makes this picture even more complex is the possibility that these processes are strictly tumor type-specific.

## Concluding Remarks

Extensive studies are being carried out to evaluate the therapeutic potential of STING agonists in combination with the next-generation cancer immunotherapies. Basic studies that will elucidate the influence of such compounds on various cell subsets that are present in tumor microenvironment are of huge importance in this process. The majority of reports indicate that these are mostly tumor stromal cells of the immune system, such as dendritic cells, that are responsible for the majority of STING agonists-mediated antitumor effects. However, it cannot be excluded that also other cells, including cancer cells, are being actively targeted by these compounds and that activation or rather inhibition of the STING pathway in those cells may play a role in achieving their full therapeutics potential. Still the role of STING-mediated signaling in cancer cells remains largely unknown and there are many questions that remain unanswered. It is not known if the downregulation of STING expression and impaired signaling in cancer cells creates survival advantage or if it is just a non-important by-product of carcinogenesis. Little is also known about the functional consequences of STING activation in cancer cells. It needs to be verified if the induction of STING-mediated signaling in cancer cells could lead directly to cancer cell death or as suggested by Gaston et al. (2016) to cytoprotection. Moreover, the molecular requirements for these effects must be clarified. Particularly required are studies elucidating the influence of STING pathway activation in cancer cells on their interactions with host immune cells. The differences between tumor types should also be explored. The insights obtained from this line of research can be useful in selecting patients that would benefit the most from STING agonist treatment as well as for development of the most effective combination treatments.

## References

[CR1] Abe T, Barber GN (2014). Cytosolic-DNA-mediated, STING-dependent proinflammatory gene induction necessitates canonical NF-κB activation through TBK1. J Virol.

[CR2] Ablasser A, Goldeck M, Cavlar T (2013). cGAS produces a 2′-5′-linked cyclic dinucleotide second messenger that activates STING. Nature.

[CR3] Ahn J, Ruiz P, Barber GN (2014). Intrinsic self-DNA triggers inflammatory disease dependent on STING. J Immunol.

[CR4] Baird JR, Friedman D, Cottam B (2016). Radiotherapy combined with novel STING-targeting oligonucleotides results in regression of established tumors. Cancer Res.

[CR5] Bhatelia K, Singh A, Tomar D (2014). Antiviral signaling protein MITA acts as a tumor suppressor in breast cancer by regulating NF-κB induced cell death. Biochim Biophys Acta.

[CR6] Chandra D, Quispe-Tintaya W, Jahangir A (2014). STING ligand c-di-GMP improves cancer vaccination against metastatic breast cancer. Cancer Immunol Res.

[CR7] Chen H, Pei R, Zhu W (2014). An alternative splicing isoform of MITA antagonizes MITA-mediated induction of type I IFNs. J Immunol.

[CR8] Civril F, Deimling T, De Oliveira Mann CC (2013). Structural mechanism of cytosolic DNA sensing by cGAS. Nature.

[CR9] Corrales L, Glickman LH, McWhirter SM (2015). Direct activation of STING in the tumor microenvironment leads to potent and systemic tumor regression and immunity. Cell Rep.

[CR10] Deng L, Liang H, Xu M (2014). STING-dependent cytosolic DNA sensing promotes radiation-induced type I interferon-dependent antitumor immunity in immunogenic tumors. Immunity.

[CR11] Ding Q, Cao X, Lu J (2013). Hepatitis C virus NS4B blocks the interaction of STING and TBK1 to evade host innate immunity. J Hepatol.

[CR12] Du M, Liu J, Chen X (2015). Casein kinase II controls TBK1/IRF3 activation in IFN response against viral infection. J Immunol.

[CR13] Fu J, Kanne DB, Leong M (2015). STING agonist formulated cancer vaccines can cure established tumors resistant to PD-1 blockade. Sci Transl Med.

[CR14] Gall A, Treuting P, Elkon KB (2012). Autoimmunity initiates in nonhematopoietic cells and progresses via lymphocytes in an interferon-dependent autoimmune disease. Immunity.

[CR15] Gaston J, Cheradame L, Yvonnet V (2016). Intracellular STING inactivation sensitizes breast cancer cells to genotoxic agents. Oncotarget.

[CR16] Grigg C, Blake Z, Gartrell R (2016). Talimogene laherparepvec (T-Vec) for the treatment of melanoma and other cancers. Semin Oncol.

[CR17] Guerra N, Tan YX, Joncker NT (2008). NKG2D-deficient mice are defective in tumor surveillance in models of spontaneous malignancy. Immunity.

[CR18] Ho SS, Zhang WY, Tan NY (2016). The DNA structure-specific endonuclease MUS81 mediates DNA sensor STING-dependent host rejection of prostate cancer cells. Immunity.

[CR19] Hu MM, Yang Q, Xie XQ (2016). Sumoylation promotes the stability of the DNA sensor cGAS and the adaptor STING to regulate the kinetics of response to DNA virus. Immunity.

[CR20] Ishikawa H, Barber GN (2008). STING is an endoplasmic reticulum adaptor that facilitates innate immune signalling. Nature.

[CR21] Ishikawa H, Ma Z, Barber GN (2009). STING regulates intracellular DNA-mediated, type I interferon-dependent innate immunity. Nature.

[CR22] Klarquist J, Hennies CM, Lehn M (2014). STING-mediated DNA sensing promotes antitumor and autoimmune responses to dying cells. J Immunol.

[CR23] Kodigepalli KM, Nanjundan M (2015). Induction of PLSCR1 in a STING/IRF3-dependent manner upon vector transfection in ovarian epithelial cells. PLoS One.

[CR24] Lam AR, Le Bert N, Ho SS (2014). RAE1 ligands for the NKG2D receptor are regulated by STING-dependent DNA sensor pathways in lymphoma. Cancer Res.

[CR25] Lau L, Gray EE, Brunette RL (2015). DNA tumor virus oncogenes antagonize the cGAS-STING DNA-sensing pathway. Science.

[CR26] Li Z, Liu G, Sun L (2015). PPM1A regulates antiviral signaling by antagonizing TBK1-mediated STING phosphorylation and aggregation. PLoS Pathog.

[CR27] Li T, Cheng H, Yuan H (2016). Antitumor activity of cGAMP via stimulation of cGAS-cGAMP-STING-IRF3 mediated innate immune response. Sci Rep.

[CR28] Liang D, Xiao-Feng H, Guan-Jun D (2015). Activated STING enhances Tregs infiltration in the HPV-related carcinogenesis of tongue squamous cells via the c-jun/CCL22 signal. Biochim Biophys Acta.

[CR29] Liu S, Cai X, Wu J (2015). Phosphorylation of innate immune adaptor proteins MAVS, STING, and TRIF induces IRF3 activation. Science.

[CR30] Liu Y, Li J, Chen J (2015). Hepatitis B virus polymerase disrupts K63-linked ubiquitination of STING to block innate cytosolic DNA-sensing pathways. J Virol.

[CR31] Ma Z, Jacobs SR, West J (2015). Modulation of the cGAS-STING DNA sensing pathway by gammaherpesviruses. Proc Natl Acad Sci USA.

[CR32] Mukai K, Konno H, Akiba T (2016). Activation of STING requires palmitoylation at the Golgi. Nat Commun.

[CR33] Ohkuri T, Ghosh A, Kosaka A (2014). STING contributes to anti-glioma immunity via triggering type-I IFN signals in the tumor microenvironment. Cancer Immunol Res.

[CR34] Ouyang S, Song X, Wang Y (2012). Structural analysis of the STING adaptor protein reveals a hydrophobic dimer interface and mode of cyclic di-GMP binding. Immunity.

[CR35] Paludan S, Bowie A (2013). Immune sensing of DNA. Immunity.

[CR36] Parkes EE, Walker SM, Taggart LE et al (2017) Activation of STING-dependent innate immune signaling by S-phase-specific DNA damage in breast cancer. J Natl Cancer Inst 109. doi:10.1093/jnci/djw199 10.1093/jnci/djw199 PMC5441301 27707838

[CR37] Ronco LV, Karpova AY, Vidal M (1998). Human papillomavirus 16 E6 oncoprotein binds to interferon regulatory factor-3 and inhibits its transcriptional activity. Genes Dev.

[CR38] Russell SJ, Peng KW, Bell JC (2012). Oncolytic virotherapy. Nat Biotechnol.

[CR39] Saitoh T, Fujita N, Hayashi T (2009). Atg9a controls dsDNA-driven dynamic translocation of STING and the innate immune response. Proc Natl Acad Sci USA.

[CR40] Shu C, Yi G, Watts T (2012). Structure of STING bound to cyclic di-GMP reveals the mechanism of cyclic dinucleotide recognition by the immune system. Nat Struct Mol Biol.

[CR41] Sun W, Li Y, Chen L (2009). ERIS, an endoplasmic reticulum IFN stimulator, activates innate immune signaling through dimerization. Proc Natl Acad Sci USA.

[CR42] Sun L, Wu J, Du F (2013). Cyclic GMP-AMP synthase is a cytosolic DNA sensor that activates the type I interferon pathway. Science.

[CR43] Sunthamala N, Thierry F, Teissier S (2014). E2 proteins of high risk human papillomaviruses down-modulate STING and IFN-κ transcription in keratinocytes. PLoS One.

[CR44] Takashima K, Takeda Y, Oshiumi H (2016). STING in tumor and host cells cooperatively work for NK cell-mediated tumor growth retardation. Biochem Biophys Res Commun.

[CR45] Takeuchi O, Akira S (2010). Pattern recognition receptors and inflammation. Cell.

[CR46] Tanaka Y, Chen ZJ (2012). STING specifies IRF3 phosphorylation by TBK1 in the cytosolic DNA signaling pathway. Sci Signal.

[CR47] Tang CH, Zundell J, Ranatunga S (2016). Agonist-mediated activation of STING induces apoptosis in malignant B cells. Cancer Res.

[CR48] Tsuchida T, Zou J, Saitoh T (2010). The ubiquitin ligase TRIM56 regulates innate immune responses to intracellular double-stranded DNA. Immunity.

[CR49] Wang Q, Liu X, Cui Y (2014). The E3 ubiquitin ligase AMFR and INSIG1 bridge the activation of TBK1 kinase by modifying the adaptor STING. Immunity.

[CR50] Wang Y, Lian Q, Yang B (2015). TRIM30α is a negative-feedback regulator of the intracellular DNA and DNA virus-triggered response by targeting STING. PLoS Pathog.

[CR51] Wang X, Teng F, Kong L (2016). PD-L1 expression in human cancers and its association with clinical outcomes. Onco Targets Ther.

[CR52] Woo SR, Fuertes MB, Corrales L (2014). STING-dependent cytosolic DNA sensing mediates innate immune recognition of immunogenic tumors. Immunity.

[CR53] Wu J, Chen ZJ (2014). Innate immune sensing and signaling of cytosolic nucleic acids. Annu Rev Immunol.

[CR54] Xia T, Konno H, Ahn J (2016). Deregulation of STING signaling in colorectal carcinoma constrains DNA damage responses and correlates with tumorigenesis article deregulation of STING signaling in colorectal carcinoma constrains DNA damage responses and correlates with tumorigenesis. Cell Rep.

[CR55] Xia T, Konno H, Barber GN (2016). Recurrent loss of STING signaling in melanoma correlates with susceptibility to viral oncolysis. Cancer Res.

[CR56] Yang C, Huang H, Chang Y (2017). DNA-sensing and nuclease gene expressions as markers for colorectal cancer progression. Oncology.

[CR57] Yi G, Brendel VP, Shu C (2013). Single nucleotide polymorphisms of human STING can affect innate immune response to cyclic dinucleotides. PLoS One.

[CR58] Yin Q, Tian Y, Kabaleeswaran V (2012). Cyclic di-GMP sensing via the innate immune signaling protein STING. Mol Cell.

[CR59] Yuen CK, Chan CP, Fung SY (2016). Suppression of type I interferon production by human T-cell leukemia virus type 1 oncoprotein Tax through inhibition of IRF3 phosphorylation. J Virol.

[CR60] Zhong B, Yang Y, Li S (2008). The adaptor protein MITA links virus-sensing receptors to IRF3 transcription factor activation. Immunity.

[CR61] Zhong B, Zhang L, Lei C (2009). The ubiquitin ligase RNF5 regulates antiviral responses by mediating degradation of the adaptor protein MITA. Immunity.

[CR62] Zhu Q, Man SM, Gurung P (2014). Cutting edge: STING mediates protection against colorectal tumorigenesis by governing the magnitude of intestinal inflammation. J Immunol.

